# Bedaquiline as Treatment for Disseminated Nontuberculous *Mycobacteria* Infection in 2 Patients Co-Infected with HIV

**DOI:** 10.3201/eid2703.202359

**Published:** 2021-03

**Authors:** Eliza Gil, Nicola Sweeney, Veronica Barrett, Stephen Morris-Jones, Robert F. Miller, Victoria J. Johnston, Michael Brown

**Affiliations:** University College London Hospitals, National Health Service Foundation Trust, London, UK (E. Gil, N. Sweeney, V. Barrett, S. Morris-Jones, V.J. Johnston, M. Brown);; Central and North West London National Health Service Foundation Trust, London (R.F. Miller);; University College London, London (R.F. Miller);; London School of Hygiene and Tropical Medicine, London (R.F. Miller, V.J. Johnston, M. Brown)

**Keywords:** bedaquiline, antibacterial drugs, antitubercular drugs, antiinfective drugs, antitubercular drugs, Mycobacteriaceae, Mycobacterium, Mycobacterium abscessus, Mycobacterium avium, nontuberculous mycobacteria, NTM, bacteria, tuberculosis and other mycobacteria, HIV, viruses, co-infection, antimicrobial resistance, respiratory infections, ^18^fluorodeoxyglucose-positron emission tomography/computed tomography imaging

## Abstract

Nontuberculous mycobacteria can cause disseminated infections in immunocompromised patients and are challenging to treat because of antimicrobial resistance and adverse effects of prolonged multidrug treatment. We report successful treatment with bedaquiline, a novel antimycobacterial drug, as part of combination therapy for 2 patients with disseminated nontuberculous mycobacteria co-infected with HIV.

Nontuberculous mycobacteria (NTM) cause a broad spectrum of disease, most commonly pulmonary infection, but also cause disseminated infection in immunocompromised patients, posing a major risk for illness and death (*1*). Treatment involves immune function optimization and prolonged use of combinations of species-specific antimycobacterial drugs but is often complicated by the intrinsic or acquired drug resistance of NTM (*2*) and adverse effects of the drug combinations; treatment failure is common. Therefore, there is considerable interest in the use of novel drugs (*3*).

Bedaquiline, a novel, oral, diarylquinolone antimycobacterial drug, is used in treatment of infections with multidrug-resistant *Mycobacterium tuberculosis* (*4*). However, its role in treatment of disseminated NTM infections remains unclear. We report the successful use of bedaquiline in treatment for 2 HIV-infected patients in London, UK, who had disseminated NTM infections.

## The Study

Case-patient 1 was a 54-year-old HIV-infected man who had colonic perforation secondary to rectal trauma. He underwent an emergency Hartmann’s procedure and showed an uncomplicated immediate recovery. Two months later, he showed development of fevers, breathlessness, and a purulent exudate at the abdominal wound site, which did not improve after receiving antimicrobial drug therapy. Imaging showed pleural effusions and perihepatic collections; mycobacterial liquid culture of effusions, collections, and wound exudate contained *M. abscessus*, presumed secondary to fecal abdominal cavity contamination. Mycobacterial blood cultures were negative.

**Figure 1:**
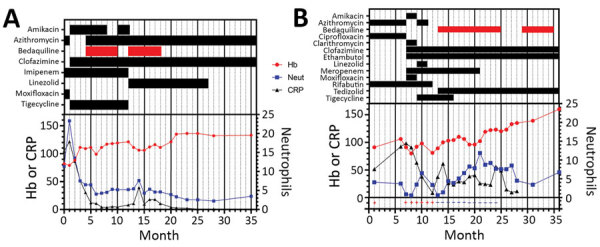
Summary of treatment and monitoring of 2 HIV-positive persons who had disseminated *Mycobacterium abscessus* infections, London, UK. A) Case-patient 1. B) Case-patient 2. The infection in case-patient 1 was secondary to fecal abdominal cavity contamination after rectal perforation. Bars in top section show timing of treatments; red indicates bedaquiline. Drug regimens and treatment responses were measured by using Hb (g/L), Neut (× 10^9^ cells/L), and CRP (mg/L). Values (+ and –) on the bottom of panel B are results for mycobacterial blood cultures. CRP, C-reactive protein; Hb, hemoglobin; Neut, neutrophils.

At diagnosis of his disseminated NTM infection, HIV viral load was undetectable (CD4 count >900 cells/μL). Empirical treatment was begun and then refined after speciation as *M. abscessus* (Figure 1, panel A)*.* Susceptibility testing subsequently demonstrated extensive drug resistance (Table 1). MIC estimations for bedaquiline showed in vitro susceptibility (MIC <0.0625 mg/L). There was no information on Clinical and Laboratory Standards Institute/European Committee on Antimicrobial Susceptibility Testing for *M. abscessus*. The MIC breakpoint for this drug with *M. tuberculosis* was 0.25 mg/L (T. McHugh, University College London, pers. comm., 2020 Jun 29). Compassionate access to bedaquiline was obtained from Janssen-Cilag https://www.janssen.com). Treatment was initiated (400 mg/d for 2 wks, followed by 200 mg 3×/wk), as per treatment for tuberculosis. Bedaquiline was well tolerated and treatment continued for a year; there was a brief interruption because of a delay in reapproval.

**Table 1:** MICs and CLSI interpretation as reported by Public Health England reference laboratory for all drugs tested against *Mycobacterium abscessus* isolate from case-patient 1 (*5*)*

Drug	MIC, mg/L	CLSI interpretation
Amikacin	Month 0: 16	Sensitive
	Month 3: 32	Intermediate
Cefoxitin	128	Resistant
Ciprofloxacin	>4	Resistant
Clarithromycin	>16	Resistant (phenotype suggestive of inducible resistance)
Cotrimoxazole	>8/152	Resistant
Doxycycline	>16	Resistant
Imipenem	16	Intermediate
Linezolid	Month 0: 32	Resistant
	Month 3: 16	Intermediate
Moxifloxacin	>8	Resistant
Tigecycline	2	No defined breakpoints
Tobramycin	8	Resistant

*MICs were obtained for the initial isolate of the patient at month 0, and again at month 3. Where duplicated, values were consistent, except for amikacin and linezolid, where both MICs are included. CLSI, Clinical and Laboratory Standards Institute.

During his treatment for NTM, the patient showed adverse effects caused by intravenous amikacin (1 g/d) and mild renal impairment. Therefore, this drug was withheld until his renal function recovered. On its reinitiation at the same dose, tinnitus developed prompting permanent withdrawal of amikacin. Because of excellent progress, tigecycline was replaced with linezolid at that time.

**Figure 2:**
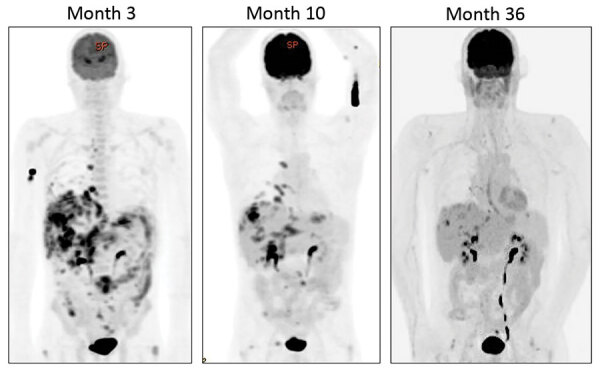
Serial ^18^fluorodeoxyglucose-positron emission tomography/computed tomography imaging quantification of disease burden for an HIV-positive person (case-patient 1) given treatment for disseminated *Mycobacterium abscessus* infection, London, UK. Images demonstrate marked reduction in fluorodeoxyglucose avidity over time.

^18^Fluorodeoxyglucose-positron emission tomography/computed tomography imaging was used to monitor disease response (Figure 2). Although pulmonary and hepatic lesions emerged intermittently, they were consistently culture negative and are believed to represent immune-mediated lesions. His most recent scan 36 months after treatment began showed ongoing, but greatly reduced, ^18^fluorodeoxyglucose avidity in all areas. Therefore, he continues maintenance therapy with clofazimine and azithromycin, despite evidence of clarithromycin resistance in vitro. This combination (clofazimine and azithromycin) has been well tolerated, and the condition of the patient continues to be favorable.

Case-patient 2 was a 30-year-old man who had pyrexia, pancytopenia, and lymphadenopathy. Advanced HIV infection had been diagnosed 1 month earlier (CD4 count 10 cells/μL, viral load >1 million copies/mL). He started antiretroviral therapy 10 days before he came to the hospital. Culture of lymph node, peripheral blood, and sputum all yielded *M. avium*. Treatment with azithromycin, rifabutin, and ciprofloxacin was initiated. Ethambutol was excluded because of color blindness.

The patient initially transferred his care to another hospital but returned 6 months later because of abdominal pain. Mycobacterial blood cultures had remained persistently positive throughout the intervening period, and at his return cultures of blood, bone marrow, and lymph node were positive for *M. avium*. We performed sensitivity testing (Table 2). Few drugs have MIC values for *M. avium*, and the correlation between MIC and clinical outcomes for drugs other than clarithromycin is unclear (*5*). The isolate demonstrated new clarithromycin resistance, postulated to have emerged because of persistence through treatment with a macrolide-containing regimen without ethambutol (*6*). His treatment was intensified by addition of amikacin and, after discussion with ophthalmologists, ethambutol. Because he continued to have fevers, meropenem, which covered the possibility of bacterial sepsis, and clofazimine were initiated, leading to symptomatic improvement. Because his symptoms worsened again on brief cessation of meropenem, it was continued.

**Table 2:** MICs and CLSI interpretation as reported by Public Health England reference laboratory for all drugs tested against *Mycobacterium abscessus* isolate from case-patient 2 (*5*)*

Drug	MIC, mg/L	CLSI interpretation
Amikacin intravenous	>64	Resistant
Ciprofloxacin	>16	No defined breakpoints
Clarithromycin	Not reported	Month 0: sensitive; month 6: high-level resistance
Doxycycline	>16	No defined breakpoints
Ethambutol	>16	No defined breakpoints
Linezolid	64	Resistant
Moxifloxacin	>8	Resistant
Rifampin	>8	No defined breakpoints

*MICs were calculated for the isolate at the initial presentation (month 0) of the patient and again at his representation 6 mo later. The second testing identified a new high level of resistance. CLSI, Clinical and Laboratory Standards Institute.

Treatment for this patient required multiple modifications because of adverse drug effects (Figure 1, panel B). Amikacin was stopped because of renal toxicity. Given the extensive in vitro resistance, best practice recommendation to add 2 drugs synchronously, lack of access to bedaquiline at this time, and evidence for a possible benefit of tigecycline in combination with a macrolide (*7*), tigecycline and linezolid were initiated in its place, causing nausea and anemia, respectively. He also had arthralgia secondary to moxifloxacin and QTc prolongation caused by azithromycin, which required their cessation.

Shortly after linezolid and azithromycin were discontinued his fevers and neutropenia returned. Mycobacterial blood cultures again showed *M. avium* despite prolonged treatment and immune reconstitution. Although bedaquiline sensitivity of this isolate was not determined in vitro, compassionate access to bedaquiline was obtained from Janssen-Cilag, and treatment was initiated at month 13 (dosing as reported for case-patient 1), along with tedizolid. Rifabutin was discontinued because of concerns over its effect on bedaquiline pharmacokinetics.

After bedaquiline and tedizolid were initiated, his fevers resolved, and he made a steady recovery and had no side effects. Given his high risk for relapse, he received bedaquiline for 18 months on the advice of the British Thoracic Society panel, ensuring a year of effective therapy since his last positive blood culture. He has successfully immune reconstituted, continues to receive only antiretroviral therapy, and remains healthy.

## Conclusions

Treating disseminated NTM infections is challenging and often complicated by antimicrobial resistance and adverse effects of combination drug therapy. In multidrug-resistant *M. tuberculosis*, bedaquiline has been shown to decrease the time to sputum culture negativity and improve outcomes (*8*), leading to interest in its use for NTM infections.

In vitro sensitivity of NTM to bedaquiline has been demonstrated (*9*,*10*), although several species, including *M. novocastrense*, *M. shimodei*, and *M. xenopi*, are intrinsically resistant (*11*). In addition, although bedaquiline is bactericidal against many mycobacterial species, it might only be bacteriostatic against *M. avium* (*12*). Despite this feature, bedaquiline has been used in salvage treatment for pulmonary infections with NTM (*13*), but little experience regarding its use for disseminated NTM infections has been published.

These 2 case-patients were given bedaquiline on compassionate grounds, given the lack of alternative options because of drug resistance and toxicity. For both patients, bedaquiline enabled construction of an antimicrobial drug regimen that included >2 drugs to which the organism was susceptible in vitro and probably contributed to their positive outcomes. Both patients tolerated the drug and made good clinical progress after its initiation. Given the need for combination therapy, it is impossible to attribute the positive outcome of these cases to a single drug. Both patients received bedaquiline and clofazimine because there is evidence that this combination is synergistic against NTMs in vitro (*14*). Case-patient 2 only achieved sustained mycobacterial culture negativity after treatment with bedaquiline and tedizolid.

Use of bedaquiline as salvage therapy for pulmonary NTM infection is often complicated by the emergence of drug resistance and disease relapse (*15*). These case-patients received bedaquiline for >1 year, and neither showed evidence of the acquisition of drug resistance or disease relapse over that time or since. These case-patients provide support for use of bedaquiline for treatment of disseminated NTM infection, particularly when standard regimens cannot be used because of drug resistance or adverse drug effects.

## References

[1] Holland SM. Nontuberculous mycobacteria. Am J Med Sci. 2001;321:49–55. 10.1097/00000441-200101000-00008 11202480

[2] Brown-Elliott BA, Nash KA, Wallace RJ Jr. Antimicrobial susceptibility testing, drug resistance mechanisms, and therapy of infections with nontuberculous mycobacteria. Clin Microbiol Rev. 2012;25:545–82. 10.1128/CMR.05030-11 22763637 PMC3416486

[3] Millar BC, Moore JE. Antimycobacterial strategies to evade antimicrobial resistance in the nontuberculous mycobacteria. Int J Mycobacteriol. 2019;8:7–21. 10.4103/ijmy.ijmy_153_18 30860173

[4] Pym AS, Diacon AH, Tang S-J, Conradie F, Danilovits M, Chuchottaworn C, et al.; TMC207-C209 Study Group. Bedaquiline in the treatment of multidrug- and extensively drug-resistant tuberculosis. Eur Respir J. 2016;47:564–74. 10.1183/13993003.00724-2015 26647431

[5] M24Ed3 susceptibility testing of mycobacteria, *Nocardia* spp., and other aerobic Actinomycetes, 3rd ed. Wayne (PA): Clinical and Laboratory Standards Institute [cited 2020 Nov 6]. https://clsi.org/standards/products/microbiology/documents/m24 31339680

[6] Schön T, Chryssanthou E. Minimum inhibitory concentration distributions for *Mycobacterium avium* complex-towards evidence-based susceptibility breakpoints. Int J Infect Dis. 2017;55:122–4. 10.1016/j.ijid.2016.12.027 28069470

[7] Benson CA, Williams PL, Currier JS, Holland F, Mahon LF, MacGregor RR, et al.; AIDS Clinical Trials Group 223 Protocol Team. A prospective, randomized trial examining the efficacy and safety of clarithromycin in combination with ethambutol, rifabutin, or both for the treatment of disseminated *Mycobacterium avium* complex disease in persons with acquired immunodeficiency syndrome. Clin Infect Dis. 2003;37:1234–43. 10.1086/378807 14557969

[8] Bax HI, Bakker-Woudenberg IA, Ten Kate MT, Verbon A, de Steenwinkel JE. Tigecycline potentiates clarithromycin activity against *Mycobacterium avium* in vitro. Antimicrob Agents Chemother. 2016;60:2577–9. 10.1128/AAC.02864-15 26883697 PMC4808172

[9] Diacon AH, Pym A, Grobusch MP, de los Rios JM, Gotuzzo E, Vasilyeva I, et al.; TMC207-C208 Study Group. Multidrug-resistant tuberculosis and culture conversion with bedaquiline. N Engl J Med. 2014;371:723–32. 10.1056/NEJMoa1313865 25140958

[10] Aguilar-Ayala DA, Cnockaert M, André E, Andries K, Gonzalez-Y-Merchand JA, Vandamme P, et al. In vitro activity of bedaquiline against rapidly growing nontuberculous mycobacteria. J Med Microbiol. 2017;66:1140–3. 10.1099/jmm.0.000537 28749330 PMC5817190

[11] Martin A, Godino IT, Aguilar-Ayala DA, Mathys V, Lounis N, Villalobos HR. In vitro activity of bedaquiline against slow-growing nontuberculous mycobacteria. J Med Microbiol. 2019;68:1137–9. 10.1099/jmm.0.001025 31210631

[12] Cholo MC, Mothiba MT, Fourie B, Anderson R. Mechanisms of action and therapeutic efficacies of the lipophilic antimycobacterial agents clofazimine and bedaquiline. J Antimicrob Chemother. 2017;72:338–53. 10.1093/jac/dkw426 27798208

[13] Lounis N, Gevers T, Van den Berg J, Vranckx L, Andries K. ATP synthase inhibition of *Mycobacterium avium* is not bactericidal. Antimicrob Agents Chemother. 2009;53:4927–9. 10.1128/AAC.00689-09 19738016 PMC2772307

[14] Alexander DC, Vasireddy R, Vasireddy S, Philley JV, Brown-Elliott BA, Perry BJ, et al. Emergence of mmpT5 variants during bedaquiline treatment of *Mycobacterium intracellulare* lung disease. J Clin Microbiol. 2017;55:574–84. 10.1128/JCM.02087-16 27927925 PMC5277528

[15] Ruth MM, Sangen JJN, Remmers K, Pennings LJ, Svensson E, Aarnoutse RE, et al. A bedaquiline/clofazimine combination regimen might add activity to the treatment of clinically relevant non-tuberculous mycobacteria. J Antimicrob Chemother. 2019;74:935–43. 10.1093/jac/dky526 30649327

